# Cerebrospinal Fluid Biomarker Candidates Associated with Human WNV Neuroinvasive Disease

**DOI:** 10.1371/journal.pone.0093637

**Published:** 2014-04-02

**Authors:** Christophe Fraisier, Anna Papa, Samuel Granjeaud, Rogier Hintzen, Byron Martina, Luc Camoin, Lionel Almeras

**Affiliations:** 1 Aix Marseille Université, Unité de Recherche en Maladies Infectieuses et Tropicales Emergentes, UM63, CNRS 7278, IRD 198, Inserm 1095, Marseille, France; 2 Unité de Recherche en Biologie et Epidémiologie Parasitaires (URBEP), Institut de Recherche Biomédicale des Armées (IRBA), Marseille, France; 3 Department of Microbiology, Medical School, Aristotle University of Thessaloniki, Thessaloniki, Greece; 4 CRCM, Marseille Protéomique, Inserm, U1068, Marseille, France; 5 Aix-Marseille Université, UM 105, Marseille, France; 6 Department of Neurology, Erasmus MC, Rotterdam, The Netherlands; 7 Department of Virology, Erasmus MC, Rotterdam, The Netherlands; University of Texas Medical Branch, United States of America

## Abstract

During the last decade, the epidemiology of WNV in humans has changed in the southern regions of Europe, with high incidence of West Nile fever (WNF) cases, but also of West Nile neuroinvasive disease (WNND). The lack of human vaccine or specific treatment against WNV infection imparts a pressing need to characterize indicators associated with neurological involvement. By its intimacy with central nervous system (CNS) structures, modifications in the cerebrospinal fluid (CSF) composition could accurately reflect CNS pathological process. Until now, few studies investigated the association between imbalance of CSF elements and severity of WNV infection. The aim of the present study was to apply the iTRAQ technology in order to identify the CSF proteins whose abundances are modified in patients with WNND. Forty-seven proteins were found modified in the CSF of WNND patients as compared to control groups, and most of them are reported for the first time in the context of WNND. On the basis of their known biological functions, several of these proteins were associated with inflammatory response. Among them, Defensin-1 alpha (DEFA1), a protein reported with anti-viral effects, presented the highest increasing fold-change (FC>12). The augmentation of DEFA1 abundance in patients with WNND was confirmed at the CSF, but also in serum, compared to the control individual groups. Furthermore, the DEFA1 serum level was significantly elevated in WNND patients compared to subjects diagnosed for WNF. The present study provided the first insight into the potential CSF biomarkers associated with WNV neuroinvasion. Further investigation in larger cohorts with kinetic sampling could determine the usefulness of measuring DEFA1 as diagnostic or prognostic biomarker of detrimental WNND evolution.

## Introduction

West Nile virus (WNV) belongs to the *Flaviviridae* family (genus *Flavivirus*), transmitted to humans by the bite of infectious mosquitoes, mainly from the *Culex* genus [1]. In nature, avian hosts are the main reservoir of WNV; nevertheless transmission to other vertebrates can occur [2]. Humans and equines are sensitive to WNV infection, but they are considered as incidental dead-end hosts, due to the low and short viremia [3].While the majority of humans infected with WNV remain asymptomatic, approximately 20% develop a transient febrile illness, known as West Nile fever (WNF); and about 1% of infected individuals develop a neuroinvasive disease (WNND) characterized by encephalitis, meningitis, and/or acute flaccid paralysis, leading in some cases to a fatal outcome [4], [5].

During the last decade, the epidemiology of WNV has changed in the more southern regions of Europe, with increased incidence of WNND in humans [6]. Unexpectedly in 2010, the first large outbreak of WNV infections occurred in Greece [7]; it was characterized by the census of nearly 200 WNND cases with 17% fatality rate [8]. Since then, the reports of WNV outbreaks are increasing. A recent update on the Italian epidemic between 2008 and 2011, reported 43 cases of WNND with 16% fatality rate [9], while in Balkans several WNND cases have been reported [10], [11], [12]. The recent epidemiologic studies underline that in Europe, like in the United States, WNV infections, and particularly WNND cases, have become of major public health concern [13]. Despite the development of successful WNV vaccines for equines and the ongoing clinical trials for human -vaccines, no licensed vaccine for human use is currently available [5]. Antibody therapy (*i.e.*, WNV-specific immunoglobulins) [14], [15], ribavirin [16], [17] and treatment with interferon-α [18], [19] showed therapeutic potential for clinical use in humans, but larger clinical trials are required to establish their therapeutic efficacy. Thus, currently no specific antiviral treatment for WNV infections is available and only supportive care is administered. Although the management of WNV infection has progressed by the development of molecular and serological tools for laboratory diagnosis [5], [20], early clinical diagnosis is still challenging because the initial symptoms are not specific. The prediction of clinical evolution to either recovery or central nervous system (CNS) involvement has not been yet elucidated. Thus, the identification of biomarkers associated with disease severity could enable a better forecasting of WNV disease progression (*i.e.*, distinction between WNND and WNF) and could improve knowledge of brain alteration to define novel potential drug targets.

The CNS is separated from the rest of the body by blood interfaces, which are mainly composed of the blood-brain barrier (BBB) and the blood-cerebrospinal fluid (CSF) barrier, which maintain the CNS homeostasis, and preserve it from toxic compounds or pathogens. However several neurotropic viruses, such as WNV, can bypass BBB and invade the CNS [21], [22]. By its close contact with the extracellular fluid of the brain, analysis of CSF composition can reflect biological CNS impairments enabling the diagnosis and understanding of various neurodegenerative CNS disorders [23]. The presence of WNV-specific IgM antibodies in the CSF is a strong evidence of a recent infection, however, neutralization assays or detection of WNV RNA could be necessary in case of suspicion infection with other flaviviruses due to cross-reactivity [24]. Furthermore, the persistence of WNV IgM antibodies in CSF for several months after the onset of the disease complicates the laboratory diagnosis [25]. Thus, these biological tests could reflect neurological infection, but not direct relationship with acute encephalitis.

The number of studies which analyze the CSF component modifications during WNV infection, or investigate the association between imbalance of CSF elements and severity of WNV infection is very limited. Some reports postulated that the number of plasma white cells in CSF could be used as an indicator of brain WNV infection [26], [27], but these findings were not confirmed by more recent studies [28], [29], suggesting that the predictive value for WNND diagnosis is low. Recently, Petzold and collaborators observed that the CSF levels of neuronal (*i.e.*, neurofilaments) and glial (*i.e.*, glial fibrillary acidic protein (GFAP) and S100B) biomarkers were elevated in WNV encephalitis patients compared to non-inflammatory controls [30]. Nevertheless, these biomarkers were also detected in CSF and sera from WNF patients, reconsidering the use of these proteins as prognostic markers of disease severity [30], [31]. The biological content of the CSF has long been used as an indicator regarding diagnoses or etiology of CNS-related symptoms and dysfunctions [32], [33]. Thus, to monitor biological effects of CNS infections, proteomic investigations of CSF have produced quite promising results in the discovery of biomarkers associated with brain infection [34], [35].

To gain a better insight into the pathophysiology of severe WNV infection in humans and to identify potential biomarkers associated with neuroinvasion, a comparative examination of CSF protein profiles between patients with WNND and control individuals with non-inflammatory symptoms was performed using a quantitative proteomic approach (iTRAQ).

## Materials and Methods

### Ethics statement

The Medical Ethical Committees of the Erasmus University Medical Centre in Rotterdam, The Netherlands, the Sint Franciscus Gasthuis in Rotterdam, The Netherlands, and the Medical Ethical Committee of Aristotle University of Thessaloniki, Greece, approved the study protocol. Serum and CSF samples used for the ELISA validation tests were both previously banked de-identified samples collected for diagnostics, or from individuals living in Marseille, France, from whom a written informed consent was obtained, and the Marseille-2 Ethical Committee approved the protocol.

### Material

All subjects included in the present study were Caucasians. Demographic and clinical data relating to these individuals are detailed in the Table 1. They were divided into the following groups:

**Table 1:** Demographic and clinical data relating to individuals included for iTRAQ or ELISA test validation.

	WN neuroinvasive disease (WNND)			WNF	non-WNV CNS infection	AH	IIH	Healthy individuals
**Abbreviated group name**	A1	A2	A3	B1	C1	C2	C3	C4
**Number of samples**	16	8	27	23	13	6	5	21
**CSF samples**	Y	Y	N	N	Y	Y	Y	N
**Sera samples**	Y	N	Y	Y	Y	N	N	Y
**iTRAQ experiments**	N	Y	N	N	N	Y	Y	N
**ELISA analysis**	Y	N	Y	Y	Y	N	N	Y
**Age±SD, years (range)**	64.7±16.4 (19–80)	71.1±11.5 (42–87)	68.6±15.4 (44–92)	70.6±8.8 (52–84)	48.4±21.7 (20–74)	35.0±12.8 (21–56)	35.7±11.3 (26–44)	45.3±11.2 (24–62)
**Gender (% Female)**	56.3	62.5	40.7	65.2	38.5	33.3	100	47.6
**Days after the onset of the symptoms (mean days ± SD)**	6.9±6.0	6.6±3.8	7.3±4.4	6.8±3.2	10.6±8.5	/	/	/

AH, acute headache; CNS, central nervous system; CSF, cerebrospinal fluid; IIH, idiopathic intracranial hypertension; iTRAQ, Isobaric tags for relative and absolute quantitation; N, No; SD, standard deviation; Y, Yes; WNF, West Nile fever; WNND, West Nile neurodegenerative diseases; WNV, West Nile virus.

**A.** The WNND group, consisted in total of 51 Greek patients (25 females). This group included the subgroup A1 (n = 16) for whom both serum and CSF samples were available; the subgroup A2 (n = 8) for whom only a CSF sample was available; and the subgroup A3, (n = 27) for whom only a serum sample was available.

**B.** The WNF group, named B1, consisting of 23 Greek patients with WNF for whom only a serum sample was available.

**C.** The control groups, consisting of the following sub-groups: C1 group, consisting of 13 Greek patients with non-WNV CNS infection for whom paired serum and CSF was available; C2 group, consisting of 6 Dutch individuals with acute headache (AH group), for whom only a CSF sample was available; C3 group, including 5 Dutch female individuals with idiopathic intracranial hypertension (IIH group), for whom only a CSF sample was available; and C4 group, consisting of 21 apparently healthy French individuals (“healthy group”). The 11 individuals from the AH and IIH groups had no neurological disorders, did not take any medication, and were considered to have normal CSF.

### Protein sample preparation

Due to the limited volume of the CSF samples, pooled CSF samples from each experimental group (*i.e.*, A2, C2 and C3 groups) were generated by mixing an equal volume (30 μL) of each sample per group. The protein concentration of each sample pool was determined by the Lowry method (DC Protein assay Kit, Bio-Rad) according to the manufacturer's instructions. The WNND group (A2 group) had higher CSF protein concentration (mean: 907 μg/mL) than the AH (mean: 479 μg/mL, C2 group) and IIH (mean: 249 μg/mL, C3 group) control groups. Each pool was vortexed for 30 seconds and then subdivided into vials containing 30 μg protein stored at −80°C until further use.

### iTRAQ labeling of CSF samples

iTRAQ is a gel-free based technique to quantify proteins from different sources in a single experiment. This technique incorporates covalently isobaric tags to enable quantitative proteomic analysis [36]. For iTRAQ labeling, replicates of CSF sample pools containing 30 μg protein were used (Table S1). Proteins were precipitated with cold acetone for 2 h at –20°C, centrifuged for 5 min at 16 000×*g*, dissolved in 20 μL of dissolution buffer, denatured, reduced, alkylated and digested with 10 μg of trypsin overnight at 37°C, following manufacturer's protocol (iTRAQ Reagent Multiplex Buffer kit, Applied Biosystems, Foster City, CA, USA) and as previously described [37]. The resulting peptides were labeled with iTRAQ reagents (iTRAQ Reagent-8Plex multiplex kit, Applied Biosystems) according to manufacturer's instructions. Before combining the samples, a pre-mix containing an aliquot of each labeled sample, cleaned-up using a ZipTip, was analyzed by MS/MS to check for peptide labeling efficiency with iTRAQ reagents and homogeneity of labeling between each sample. The content of each iTRAQ reagent-labeled sample was then pooled into one tube according to this previous test. The mixture was then cleaned-up using an exchange chromatography (SCX/ICAT cation exchange cartridge, AB Sciex, Foster City, USA) and reverse-phase chromatography C18 cartridge (C18 SpinTips, Protea bio, Nîmes, France) prior to separation using an off-gel system (Agilent 3100 OFFGEL fractionator, Agilent Technologies), as previously described [38].

### Mass spectrometry analysis

For nanoLC mass spectrometry (MS) measurements, approximately 5 μg of peptide sample was injected onto a nanoliquid chromatography system (UltiMate 3000 Rapid Separation LC (RSLC) systems,Dionex, Sunnyvale, CA). After pre-concentration and washing of the sample on a Dionex Acclaim PepMap 100 C18 column (2 cm×100 μm internal diameter (id) 100 A, 5 μm particle size), peptides were separated on a Dionex Acclaim PepMap RSLC C18 column (15 cm×75 μm id, 100 A, 2 mm particle size) (Dionex, Amsterdam) using a linear 180 min gradient (4-40% acetonitrile/H20; 0.1% formic acid) at a flow rate of 300 nL/min. The separation of the peptides was monitored by a UV detector (absorption at 214 nm). The nanoLC was coupled to a nanospray source of a linear ion trap Orbitrap mass spectrometer (LTQ VelosOrbitrap, Thermo Electron, Bremen, Germany). The LTQ spray voltage was 1.4 kV and the capillary temperature was set at 275°C. All samples were measured in a data dependent acquisition mode. Each run was preceded by a blank MS run in order to monitor system background. The peptide masses were measured in a survey full scan (scan range 300–1700 m/z, with 30 K FWHM resolution at m/z = 400, target AGC value of 10^6^ ions and maximum injection time of 500 ms). In parallel to the high-resolution full scan in the Orbitrap, the data-dependent HCD scans of the 10 most intense precursor ions were fragmented and measured in the orbitrap analyser (normalized collision energy of 35%, activation time of 10 ms target AGC value of 10^4^, maximum injection time 100 ms, isolation window 2 Da and wideband activation enabled). Dynamic exclusion was implemented with a repeat count of 1 and exclusion duration of 30 sec.

### Data Analysis

Raw files generated from MS analysis were combined and processed with Proteome Discoverer v 1.3 (Thermo Fisher Scientific, Waltham, MA, USA). Protein identification and quantification were carried out using the Mascot search engine (v.2.3; Matrix Science, Boston, MA, USA) and SEQUEST (v.28.0.0.0; University of Washington) through Proteome Discoverer v 1.3 (Thermo Scientific). The search was performed against the *Homo Sapiens Sapiens* database containing 20257 sequences (from SwissProt, May 24^rd^, 2012). Data were processed as described previously [39]. Several comparisons were performed including WNND group (A2 group) vs AH+IIH group (C2+C3 groups), but also WNND (A2 group) vs AH (C2 group) or vs IIH (C3 group) groups. Conversely, the proteins differentially expressed between AH (C2 group) and IIH (C3 group) groups were excluded from the analysis to take into account only the protein abundance specifically altered in WNND samples. Proteins whose expression were significantly differentially expressed (|fold-change|≥2, *p*-value≤0.05) were selected for the analysis. Change in protein expression (between two groups) is reported as ratio or as fold-change (FC). Fold-change equals ratio when ratio is larger than 1; FC equals minus one over ratio when ratio is lower than 1.

### Ingenuity pathway analysis

The functional analyses were generated through the use of Ingenuity Pathways Analysis software, Inc. (http://www.ingenuity.com). In this analysis, the average relative proteins expression values (fold-change) obtained from MS analysis were uploaded into the IPA software. The IPA program uses a knowledgebase (IPA KB) derived from the scientific literature to connect genes or proteins based on their relationships and functions. IPA generates biological networks, canonical pathways and functions that are relevant to the uploaded dataset. A right-tailed Fisher's exact test is used for calculating *p*-values to determine if the probability that the association between the proteins in the dataset and the functional and canonical pathway can be explained by chance alone. The scores derived from a *p*-value (score  =  -log (*p*-value)) and indicated the likelihood that the proteins of interest (*i.e.*, the identified proteins within a network) were clustered together.

### ELISAs

Defensin alpha-1 (DEFA1) concentration was evaluated in paired CSF and serum (A1 and C1 groups) or only in serum samples from individuals of the different clinical groups (A3, B1 and C3 groups) by capture enzyme-linked immunosorbent assay (ELISA) kits according to the manufacturer's instructions (Uscn, Life Science Inc., Wuhan, China). Plates were read in an ELISA microplate reader (Versa Max TurnableMultiplate Reader, Molecular Devices, UK). Paired CSF/serum samples from the same individual were tested on the same plate in duplicate.

### Statistical analysis

Since values in each group did not follow a Gaussian distribution or sample size is too small, we used non-parametric tests. The Kruskal-Wallis test was used for multiple group comparisons. Two independent groups were compared with Mann-Whitney U test. Spearman's rank correlation coefficient was computed when appropriate. Threshold values were determined with the use of receiver operating characteristic (ROC) curve. All differences were considered significant at *p*<0.05 and statistical analyses were performed using the statistical software Prism 5 (GraphPad, Inc).

## Results

### Differential expression of CSF proteins between WNND patients and non-infected individuals

CSF samples were tested for the identification of differentially expressed proteins between WNND patients (*i.e.*, A2 group) and the control groups (*i.e.*, C2 and C3 groups), using iTRAQ labeling and MS analysis. The application of a global False Discovery Rate (FDR) of 5% gave rise to a total of 470 identified and quantified proteins that were included in the analysis.

The comparison of protein abundance variations between the AH- and IIH-control groups indicated that 11 proteins were significantly differentially expressed (average ratios≤0.5 or ≥2, *p*-value≤0.05; Table 2): six proteins were up-regulated (fold-change (FC) ranging from 2.1 to 3.5), including four proteins from the apoliprotein family (APOC1, APOB, APOL1, APOM), involved in lipoprotein particle composition responsible for lipid trafficking and metabolism, and five proteins, all belonged to the keratin protein family, were down-regulated (FC ranging from −8.8 to −2.0) (Table 2). Since these keratin proteins are mainly structural constituents of epidermis, it is possible that their up-representation in the IIH-group could result from a contamination associated with CSF sampling and handling. Thus, the protein changes concerned mainly two protein family groups, which could be attributed to sample contamination or abundance of lipoprotein particles.

**Table 2:** CSF proteins differentially expressed between the AH- and IIH-control groups (*i.e.*, C2 and C3 groups) identified by quantitative LC-MS/MS analysis.

Protein description	Accession n° (UniprotKB)	Protein Symbol	Number of unique peptides	Number of peptides	Average ratios AH/IIH^a^	Subcellular location	Biological function^b^
Apolipoprotein C-I	P02654	APOC1	4	4	2.062	Secreted	Lipid transport
Apolipoprotein B-100	P04114	APOB	80	80	2.231	Secreted	Lipid transport
Apolipoprotein L1	O14791	APOL1	6	6	2.458	Secreted	Lipid transport
Apolipoprotein M	O95445	APOM	3	3	2.379	Secreted	Lipid transport
CD5 antigen-like	O43866	CD5L	8	8	3.518	Secreted	Apoptotic process
Serum amyloid P-component	P02743	APCS	4	4	2.332	Secreted	Metal ion binding
Keratin, type I cuticular Ha1	Q15323	KRT31	3	4	0.114	Cytoskeleton	Cell organization
Keratin, type I cytoskeletal 9	P35527	KRT9	22	24	0.328	Cytoskeleton	Cell organization
Keratin, type II cuticular Hb2	Q9NSB4	KRT82	2	3	0.122	Cytoskeleton	Cell organization
Keratin, type II cuticular Hb3	P78385	KRT83	13	14	0.160	Cytoskeleton	Cell organization
Keratin, type II cytoskeletal 1	P04264	KRT1	26	30	0.498	Cytoskeleton	Cell organization

a AH : acute headache; IIH : idiopathic intracranial hypertension.

b according to Gene Ontology.

**Figure 1:**
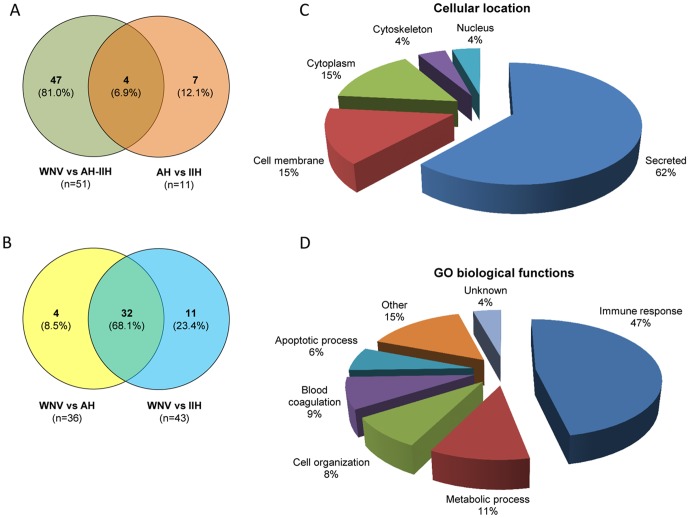
Classification of human CSF proteins significantly differentially expressed between WNND patients (*i.e.*, A2 group) and control individuals (*i.e.*, C2 and C3 groups) identified by quantitative LC-MS/MS analysis. (A) The number of proteins significantly differentially expressed between WNND group and AH+IIH control group or between AH and IIH control sub-groups are represented in the Venn diagram. (B) Venn diagram representation of proteins significantly differentially expressed between WNND group and AH or IIH control sub-groups, among the proteins found deregulated exclusively in patients with WNND compared to AH+IIH group (n = 47). The two groups that were compared are indicated below each circle. The number and the percentage of proteins associated with each category are indicated in brackets. Classification of the significantly differentially regulated proteins according to their sub-cellular location (C) and their functional categorization (D) according to gene ontology (GO). The percentages of proteins associated with each category are indicated. WNND, West Nile neuroinvasive disease; AH, acute headache; IIH, idiopathic intracranial hypertension; CSF, cerebrospinal fluid.

The comparison of protein abundance variations between WNND and the AH+IIH control groups highlighted 51 proteins that were found significantly differentially expressed (Figure 1A). Among them, 47 proteins modified exclusively in patients with WNND compared to AH+IIH group were taken into account (Figure 1B, Table 3). The large majority of these proteins were up-regulated (42 and five proteins up- and down-regulated, respectively), and fold-changes were much higher for up- (ranging from 2.0 to 12.9) than for down-regulated proteins (ranging from −2.0 to −2.2). Interestingly, 32 out of the 47 differentially expressed proteins were found commonly modified considering WNND *vs* AH and WNND *vs* IIH comparisons (Figure 1B, Table 3). The 47 significantly differentially expressed proteins were classified according to their cellular distribution and were grouped into functional categories according to gene ontology (GO) (Figure 1C and 1D), using Proteome Discoverer software 1.3 (Therome scientific) retrieving GO database information from ProteinCenter software (Thermo Scientific). As expected, a large majority of them (62%) was secreted (biological fluid) and the first functional category corresponded to immune response (47%). Among the proteins from the immune response, half of them corresponded to immunoglobulin chains (n = 10) which reflected the absence of immunoglobulin depletion prior the quantitative protein repertoire analysis, but also indicated the inflammatory phenomenon which occurred in the WNND group compared to control group.

**Table 3:** CSF proteins differentially expressed between WNND (*i.e.*, A2 group) and AH- and IIH-control groups (*i.e.*, C2 and C3 groups) identified by quantitative LC-MS/MS analysis.

Protein description	Accession n° (UniprotKB)	Protein Symbol (IPA)	Number of unique peptides	Number of peptides	Average ratios			Subcellular location	Biological function^b^
					WNND/AH+IIH^a^	WNND/AH	WNND/IIH		
Neutrophil defensin 1	P59665	DEFA1	3	3	12.894	12.178	12.213	Secreted	Immune response
Hemoglobin subunit beta	P68871	HBB	6	11	6.671	6.113	8.743	Cytoplasm	Oxygen transport
Protein S100-A9	P06702	S100A9	6	6	4.806	4.749	5.427	Cytoplasm	Immune response
Neutrophil gelatinase-associated lipocalin	P80188	LCN2	3	3	4.601	4.567	4.718	Secreted	Immune response
Protein S100-A8	P05109	S100A8	5	5	4.436	4.204	4.949	Secreted	Immune response
Hemoglobin subunit alpha	P69905	HBA*	6	6	4.129	3.766	5.597	Cytoplasm	Oxygen transport
Ig mu chain C region	P01871	IGHM	19	19	3.667	3.314	5.155	Secreted	Immune response
Plastin-2	P13796	LCP1	2	3	3.615	3.441	5.014	Cytoskeleton	Immune response
Histone H4	P62805	HIST1H4A	3	3	3.477	3.534	3.895	Nucleus	DNA binding
Spondin-1	Q9HCB6	SPON1	2	2	3.249	2.864	4.873	Secreted	Cell adhesion
Chitinase-3-like protein 1	P36222	CHI3L1	15	15	3.173	3.276	3.145	Secreted	Immune response
Hemoglobin subunit delta	P02042	HBD	4	9	2.91	2.786	3.285	Cytoplasm	Blood coagulation
Histone H2B type 1-K	O60814	HIST1H2BK	2	2	2.907	2.611	4.048	Nucleus	DNA binding
Ig kappa chain V-I region Ni	P01613	KV121*	2	2	2.892	2.851	3.097	Secreted	Immune response
Immunoglobulin J chain	P01591	IGJ	5	5	2.877	2.759	3.744	Secreted	Immune response
Ig kappa chain V-III region Ti	P01622	KV304*	2	6	2.803	2.565	2.367	Secreted	Immune response
Myeloblastin	P24158	PRTN3	2	2	2.765	2.825	2.645	Cell membrane	Metabolic process
Carbonic anhydrase 1	P00915	CA1	5	5	2.738	2.551	3.748	Cytoplasm	Metabolic process
Carbohydrate sulfotransferase 15	Q7LFX5	CHST15	2	2	2.725	2.753	2.677	Cell membrane	Metabolic process
Peroxiredoxin-2	P32119	PRDX2	3	3	2.635	2.564	2.847	Cytoplasm	Apoptotic process
Actin, cytoplasmic 1	P60709	ACTB	14	14	2.596	2.531	2.567	Cytoskeleton	Cell organization
Fibrinogen alpha chain	P02671	FGA	21	21	2.556	2.480	2.773	Secreted	Blood coagulation
IgGFc-binding protein	Q9Y6R7	FCGBP	39	39	2.516	2.615	2.216	Secreted	Unknown
Cartilage oligomeric matrix protein	P49747	COMP	2	2	2.478	2.246	3.353	Secreted	Apoptotic process
Fibrinogen gamma chain	P02679	FGG	18	18	2.46	2.457	2.401	Secreted	Blood coagulation
Monocyte differentiation antigen CD14	P08571	CD14	10	10	2.389	2.439	2.425	Cell membrane	Immune response
Ig kappa chain V-I region EU	P01598	KV106*	1	3	2.372	2.396	2.399	Secreted	Immune response
Lysozyme C	P61626	LYZ	5	5	2.365	2.407	2.312	Secreted	Immune response
Haptoglobin	P00738	HP	12	23	2.194		2.787	Secreted	Immune response
Ig alpha-1 chain C region	P01876	IGHA1	6	15	2.182	2.097	2.505	Secreted	Immune response
Ig lambda chain V-I region NIG-64	P01702	LV104*	3	3	2.165		3.096	Secreted	Immune response
Low affinity immunoglobulin gamma Fc region receptor III-A	P08637	FCGR3A	2	2	2.14	2.289		Secreted	Immune response
Matrix Gla protein	P08493	MGP	2	2	2.135		3.343	Secreted	Cell organization
Ig lambda-2 chain C regions	P0CG05	IGLC2	4	9	2.114	2.000	2.473	Secreted	Immune response
High affinity cAMP-specific and IBMX-insensitive 3′,5′-cyclic phosphodiesterase 8B	O95263	PDE8B	1	2	2.078	2.344		Cytoplasm	Metabolic process
Moesin	P26038	MSN	2	2	2.071		2.354	Cell membrane	Cell organization
Ig lambda chain V-I region WAH	P04208	LV106*	2	2	2.055		2.513	Secreted	Immune response
V-set and immunoglobulin domain-containing protein 4	Q9Y279	VSIG4	5	5	2.042	2.032	2.080	Cell membrane	Immune response
Alpha-1-antichymotrypsin	P01011	SERPINA3	19	19	2.036	2.015	2.099	Secreted	Immune response
Fibrinogen beta chain	P02675	FGB	22	22	2.035		2.132	Secreted	Blood coagulation
Metalloproteinase inhibitor 1	P01033	TIMP1	5	5	2.029	2.212		Secreted	Apoptotic process
Ig alpha-2 chain C region	P01877	IGHA2	2	11	2.019		2.373	Secreted	Immune response
Neurexin-3-alpha	Q9Y4C0	NRXN3	9	12	0.5		0.422	Cell membrane	Nervous system development
Phosphoinositide-3-kinase-interacting protein 1	Q96FE7	PIK3IP1	3	3	0.488		0.367	Cell membrane	Metabolic process
Secretogranin-1	P05060	CHGB	27	27	0.481	0.493		Secreted	Unknown
Sialate O-acetylesterase	Q9HAT2	SIAE	2	2	0.468		0.291	Secreted	Catalytic activity
Apolipoprotein E	P02649	APOE	26	26	0.465		0.364	Secreted	Lipid transport

* Proteins not mapped into IPA system.

a WNND: West Nile neuroinvasive disease; AH : acute headache; IIH : idiopathic intracranial hypertension.

b according to Gene Ontology.

### Protein networks and biological functions altered in the CSF of WNND patients

To determine significant networks and biological functions associated with human WNND cases, the 47 differentially expressed proteins identified in CSF were uploaded into the IPA system. Among them, 41 proteins were mapped in the system (Table 3). The functional analyses generated through the IPA system identified direct and indirect relationships among the proteins differentially expressed and allowed to identify two relevant networks (Table 4). These networks contained at least 15 focus molecules involved in functions related mainly to inflammatory and immunological diseases. In addition, among the biological functions related to these proteins, 15 differentially expressed proteins (DEFA1, S100A9, LCN2, S100A8, IGHM, HIST1H2BK, IGJ, PRDX2, ACTB, FGA, CD14, HP, FCGR3A, TIMP1, APOE), classified in infectious disease, were significantly associated with viral infection (*p*-value: 2.58E-05). With the exception of APOE, all these proteins were up-regulated.

**Table 4:** IPA-generated networks of differentially expressed proteins identified by iTRAQ labeling between CSF samples from WNND (*i.e.*, A2 group) and AH- and IIH-control groups (*i.e.*, C2 and C3 groups).

Top Functions	Score	Focus Molecules	Molecules in Network
Inflammatory Disease, Connective Tissue Disorders, Skeletal and Muscular Disorders	38	17	*up-regulated*
			DEFA1 (includes others), FCGR3A, FGA, FGB, FGG, HP, LCN2, LYZ, MGP, PRTN3, S100A8, S100A9, SERPINA3,
			SIAE, TIMP1
			*down-regulated*
			APOE, CHGB
			*not in the dataset*
			Alpha catenin, chymotrypsin, Collagen(s), ERK1/2, Fibrin, Fibrinogen, HDL, Ige, IL1, IL17R, Immunoglobulin, Integrin, Laminin, LDL, NADPH oxidase, Pro-inflammatory Cytokine, Stat3-Stat3, trypsin
Immunological Disease, Hematological Disease, Metabolic Disease	35	15	*up-regulated*
			ACTB, CD14, CHI3L1, FCGBP, HBB, HBD, HIST1H4A (includes others), IGHA1, IGHM, IGJ, LCP1, MSN, PDE8B,
			PRDX2,
			*down-regulated*
			PIK3IP1,
			*not in the dataset*
			Akt, caspase, CD3, chemokine, ERK, F Actin, hemoglobin, Hsp70, Iga, IgG, Igm, IL12 (complex), Insulin, Mapk, NFkB (complex), P38 MAPK, PI3K (complex), Pkc(s), RNA polymerase II, Vegf

AH : acute headache; IIH : idiopathic intracranial hypertension.

### Verification by ELISA of the selected candidate, DEFA1

Defensin alpha-1 (DEFA1) abundance in biological fluids (*i.e.*, CSF and serum) was assessed using commercial ELISA kits. Selection of DEFA1 was made on the basis of its high fold-change, the possible functional association of this protein with WNV pathobiology or the potential use as severe infection biomarker, and the availability of the required ELISA kits.

Due to their insufficient remaining volume, CSF samples from individuals previously used for the proteome repertoire comparisons could not be further assessed by ELISA (*i.e.*, A2, C2 and C3 groups). Thus, the validation tests were carried out on the WNND group (n = 16, A1 group) and the non-WNV CNS infection control group (n = 13, C1 group). Additionally to the validation of the protein abundance variations observed at the central level (*i.e.*,CSF), counterpart sera collected at the same time point were used to determine whether the selected differentially expressed protein could also be detected at the peripheral level (*i.e.*,serum). In the case of this protein would be specifically associated with neurological symptoms of WNV infection, its detection at the peripheral level could be useful and much easier for the physicians to follow and predict risk of WNV severe/detrimental evolution.

**Figure 2:**
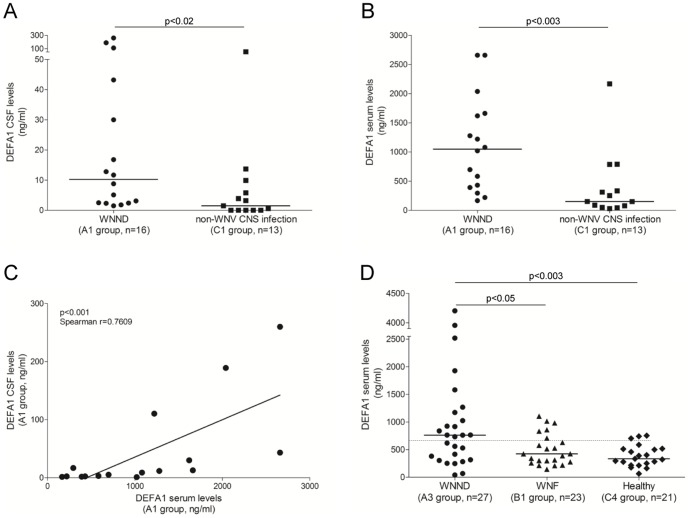
DEFA1 expression levels from patients with WNND, WNF and control groups. CSF (A) and serum (B) levels of DEFA1 measured by ELISA between WNND patients (n = 16, A1 group) and subjects with non-WNV CNS infection (n = 13, C1 group). Each dot shows the mean DEFA1 concentration detected in serum from a single individual. Horizontal bars show medians. Data were compared using the non parametric Mann-Whitney U test. (C) Correlation of DEFA1 levels between paired CSF and serum from WNND patients (n = 16, group A1). The data were analyzed using Spearman's rank correlation test with the best-fit line shown (black line). (D) Serum levels of DEFA1 measured by ELISA between WNND (n = 27, A3 group), WNF (n = 23, B1 group) patients and healthy individuals (n = 21, C4 group). Each dot shows the mean DEFA1 concentration detected in serum from a single individual. Horizontal bars show medians. Data were compared using the non parametric Mann-Whitney U test. The dotted line corresponds to the threshold value defined by ROC curve. WNND, West Nile neuroinvasive disease; WNF, West Nile fever; CNS, central nervous system; CSF, cerebrospinal fluid; DEFA1, defensin-1 alpha.

DEFA1 showed a significant increase of protein abundance in both CSF (p<0.02, Mann-Whitney U test) and sera (p<0.003, Mann-Whitney U test) from WNND patients compared to control subjects (Figure 2A and 2B). The concentration of DEFA1 in CSF (mean ± SD: 43.84±76.70 ng/mL) and serum (1.126±0.812 mg/mL) from WNND patients were found with a 6.1- and a 3.4-fold increase, respectively, than that detected in CSF (7.12±14.73 ng/mL) and serum (0.403±0.590 mg/mL) from control subjects. The CSF proteomic analysis indicated that DEFA1 was approximately 12 times more abundant in the WNND group compared to the AH+IIH group (Table 3), which is consistent with the DEFA1 ELISA test validation. Interestingly, a significant positive correlation coefficient (rho>0.76, p<0.001, Spearman's rank test) was obtained for the concentration of DEFA1 between sera and CSF from WNND patients (Figure 2C). The high levels of DEFA1 in sera from WNND group, more than 25-fold compared to counterpart CSF, led to a best-fit line running below the diagonal (slope: 0.0643±0.0183).

### Comparison by ELISA of serological DEFA1 levels between WNND patients, WNF patients and healthy controls

Finally, to assess the potential use of this protein to distinct severity of the WNV infections, the abundance of DEFA1 was measured by ELISA in serum samples from a third group of WNND patients (*i.e.*, A3 group), compared to WNF patients (*i.e.*, B1 group) and healthy controls (*i.e.*, C4 group). A significant difference was observed between the three groups (p<0.008, Kruskal-Wallis test). The level of DEFA1 was found significantly more elevated in the group of WNND patients (mean ± SD: 1.013±1.048 mg/mL) compared to the group of WNF patients (mean ± SD: 0.503±0.287 mg/mL, p<0.05, Mann-Whitney U test) and to the group of healthy individuals (mean ± SD: 0.388±0.196 mg/mL, p<0.003, Mann-Whitney U test). In contrast, despite a higher level of DEFA1 in the group of WNF patients compared to the group of healthy individuals, no significant difference could be detected (p>0.05, Mann-Whitney U test). The application of a ROC curve allowed defining the threshold value to distinguish WNND from WNF patients at the serum level (0.668 mg/mL). Using this DEFA1 threshold value, a sensitivity and specificity of 55.6% and 78.3% at the serum level were obtained, respectively.

## Discussion

The recent increasing number of WNND cases reported in Europe, the incomplete understanding of the factor(s) underlying neurological symptoms, and the lack of human vaccine or specific treatments against WNV infection impart a pressing need to characterize indicators associated with these neurological cases. Until now, no biologic marker (no laboratory marker) is available to predict the progression to WNND or to distinguish between WNF and WNND, creating diagnosis dilemmas and impeding research into understanding the pathogenesis of the disease. By its intimacy with CNS structures, any changes in the CSF composition could accurately reflect CNS pathological processes offering a unique window to study CNS disorders. Despite the importance of WNV as a CNS pathogen, there is no detailed study of CSF protein variation contents in WNND patients. Thus, the present study was conducted to investigate differences in the CSF proteome between WNND patients (*i.e.*, A2 group) and individuals with non-infectious illness (*i.e.*, C2 and C3 groups), using the iTRAQ technology. Forty-seven proteins were found exclusively modified in the CSF of WNND patients as compared to these control groups. On the basis of their known biological functions, a majority of these proteins, which are reported for the first time in WNND patients, can be associated with neurological inflammation. Some of them were proposed as potential prognostic biological markers of detrimental disease evolution/disease severity.

Among the proteins found differentially modified, 22 were classified as related to immune response with half of them corresponding to immunoglobulin chains (n = 10). The detection of immunoglobulin chains was expected because it is well known that an antibody response was elicited following WNV infection which is responsible for the clearing of the virus [40]. The presence of specific IgM antibodies against WNV antigens in CSF is currently used as diagnostic criteria of neurological involvement [5]. However, since anti-WNV IgM could persist in CSF during several months [25], and since cross-recognition with related flaviviruses could occur, other criteria should be taken into account for a definitive diagnosis [5]. Interestingly, as anti-WNV IgG have been reported to be detectable 8 days after the onset of symptoms, and since the mean time of sample collection for the WNND group was less than 7 days after illness onset, it is not surprising to identify mainly alpha (IgA) or mu (IgM) immunoglobulin heavy chains, which were found up-regulated, and reported to generally appear between 4–7 days after WNV exposure [41]. Anti-WNV IgA has been already evoked as a marker of WNV infection in CSF [42], but like anti-WNV IgM, these immunoglobulins are still detectable several months after the WNV infection [43]. Nevertheless, the up-regulation of IgM and IgA immunoglobulin heavy chains at the early stage following WNV infection, confirmed the local production of antibodies.

Additionally, immunoglobulins are one of the most abundant proteins in CSF. These proteins, together with other high abundant proteins (*e.g.*, albumin), are generally eliminated from CSF samples for proteomic analysis to reduce protein masking, allowing to increase the efficiency of low abundant protein identification [44].

A hallmark of the present study is that, at the exception of the presynaptic Neurexin-3-alpha and the floor plate-secreted Spondin-1, no other brain- or nervous system-specific proteins were found modified in WNND patients. As the aim of the present work was the characterization of potential biological marker(s) which could be further used for the diagnosis of WNND, or for the prediction of the risk to develop these severe neurological symptoms, the identification of brain-specific proteins corresponding to a neuronal damage was one of our expectations. It is probable that some proteins with weak expression level could not be detected in the present study due to the non-depletion of high abundant proteins, and then their variations in protein abundance were not considered. For example, the glial (*i.e.*, GFAP and S100B) and neuronal (*i.e.*, neurofilaments) proteins, previously detected with increased amount in CSF from WNV encephalitis patients could not be confirmed here [30].

Among the modified proteins, DEFA1 was the CSF protein with the highest abundance increase in WNND patients compared to control groups, with a FC higher than 12 (FC = +12.894). The increase of DEFA1 was even nearly twice more important than that observed for the protein showing the second highest variation (*i.e.*, Hemoglobin subunit beta, FC = +6.671). DEFA1 is a peptide composed of 30 amino acids (3.5 kDa) secreted mainly by neutrophils [45], [46]. Although α-defensins were initially identified for their antimicrobial activity, these small peptides were shown to be also active against a wide range of fungi, parasites and viruses [47], [48]. Effectively, it has been demonstrated that DEFA1 could protect against infection with some enveloped virus (*i.e.*, CMV, HSV, influenza virus) [49]. Although the precise anti-viral effect of DEFA1 is still unresolved, an inhibition of viral protein synthesis was reported [50]. To our knowledge, it is the first time that this peptide is evoked in WNV infection and its high abundance increase in WNND CSF could reflect a strong host response contributing to virus infection reduction. The up-regulation of DEFA1 was validated by ELISA at both CSF and serum levels using paired CSF/serum samples from another group of WNND patients (*i.e.*, A1 group), as compared to controls (*i.e.*, C1 group). Although the concentration of DEFA1 was 25 times more elevated in sera than in CSF of WNND patients, a positive significant correlation was observed between the two biological fluids. The detection of high serum DEFA1 concentration, more likely suggests a production of this protein at the peripheral level rather than a leak from the CSF to serum following a BBB disruption. Interestingly, it is not uncommon to detect increased number of neutrophils in the CSF of WNND cases [27], [51], suggesting that DEFA1 detected in CSF could result from a release of CSF neutrophils. It will be, thus, interesting to determine whether there is a relationship between DEFA1 abundance and CSF neutrophils count in the CSF, and to investigate the role of DEFA1 in the WNND physiopathology. Nevertheless, based on the present results, it could not be excluded that the CSF DEFA1 was produced by local neutrophils or from production in serum.

Moreover, a confirmation of significant higher serum DEFA1 levels was noticed using another group of WNND patients (*i.e.*, A3 group) compared to healthy individuals (*i.e.*, C4 group) but also to WNF patients (*i.e.*, B1 group). The higher abundance of DEFA1 at the peripheral level in WNND patients compared to those with WNF, suggests that this peptide could serve as potential biologic marker of WNV neuroinvasion. Nevertheless, the specificity of this assay should be confirmed in larger cohorts of WNND and WNF patients in a blind study. Additionally, cross checking experiments in CSF and/or serum samples from patients infected with other neurotropic viruses (*e.g.*, herpesviruses, enteroviruses) are needed to determine whether the DEFA1 release is specific for WNV or it is a common host response in viral CNS infections. However, the confirmation of this assay to distinguish WNND from WNF cases is the first demonstration of a laboratory biomarker which could enable physicians to more rapidly diagnose patients with neuroinvasive disease.

*In silico* analysis highlighted that 15 differentially expressed proteins were significantly associated with viral infection. Among them, LCN2, TIMP1, S100A8/9, and PRDX2 related to host response and/or inflammation in neurological disorders, could be used as potential biomarkers of brain pathology. Neutrophil gelatinase-associated lipocalin (LCN2) is expressed in neutrophils and is involved in innate immunity. LCN2 is expressed and secreted under inflammatory conditions and up-regulates chemokines expression in the CNS, particularly CXCL10 by astrocytes [52]. Neurodegenerative diseases are characterized by a glial reaction and LCN2 has been shown to be secreted by reactive astrocytes as a neurotoxic mediator leading to neuron death [53]. LCN2 was also reported to be increased in CSF and plasma from patients with multiple sclerosis [54]. The identification of LCN2 with an increased expression in the CSF from WNND patients (FC>4.0) in the present study could be the result from its expression in choroid plexus (CP)-infiltrating neutrophils during the acute phase of inflammation, as previously described [55], [56]. LCN2 could also bind the matrix metalloproteinase MMP9 to stabilize its proteolytic activity and prevent its degradation [57]. MMPs are responsible of BBB disruption by degrading the extracellular matrix, leading to cell invasion and cytokine production [58]. In parallel, tissue inhibitors of metalloproteinases (TIMPs) inhibit MMPs but paradoxically may also contribute to the inflammatory process by keeping a balance between deposition and degradation of extracellular proteins. This could be illustrated by the increased levels of MMPs (including MMP9) and TIMP1 in CSF from subjects with cerebral adrenoleukodystrophy (cALD) which causes neuroinflammation and demyelination [59]. Interestingly, TIMP1 was found to be increased in our study in WNND patients [60], and could reflect brain damages following WNV neuroinvasion. The role of TIMP1 in neuroinflammation was moreover supported by the gene up-regulation in Venezuelian equine encephalitis virus (VEEV)-infected mouse brain [61].

Interestingly, two members of the S100 family proteins, S100A8 and S100A9, were both found up-regulated in patients with WNND, with a FC>4.0. S100A8 and S100A9 proteins are considered damage-associated molecular pattern molecules (DAMPs) [62], constitutively expressed in neutrophils and inducible in several inflammatory cells including macrophages [63]. They are released in response to tissue injury and play an important function in innate immunity by binding to Toll-Like Receptor 4 (TLR-4) [64].

An increase abundance of PRDX2 was also detected in CSF from WNND patients (FC>2.5). Peroxiredoxins have been recently reviewed as biomarkers of oxidative stress [65], which is considered one cause of pathogenic mechanisms in neurological diseases. In particular, oxidation of PRDX2 was described to occur in pathological situations and inflammation where neutrophils are activated [66]. PRDX2 has been reported contributing to neuroprotection and was proposed for treatment for neurodegenerative diseases [67]. To our knowledge, this is the first time that PRDX2 was reported in CSF from patients with viral neuroinvasive disease. Altogether, the elevated levels of PRDX2 and S100A8/9 seem to indicate a host response to CNS infection in WNND patients and further evaluation of these proteins as relevant markers of severe WN disease would be needed.

## Conclusion

The present study represents the first analysis of CSF proteome from patients with WNND, enabling the identification of a list of protein changes which can be helpful in the better understanding of the WNV pathophysiology. Besides expected immunoglobulins, several proteins were of particular interest as being associated with host response and inflammation in neurological disorders. The identification of potential biological biomarker(s), such as DEFA1, could enable physicians to diagnose more rapidly the neurological involvement. Furthermore, additional neutrophil-related proteins (LCN2, TIMP1, S100A8/9, PRDX2) were found at higher levels in CSF of WNND patients, underlining the likely key role played by neutrophils in the development of the inflammatory response and brain damage. Further investigation is warranted to determine the usefulness of these proteins, like DEFA1, as a diagnostic tool which can be followed-up in monitoring the patients with neuroinvasive disease.

## Supporting Information

Table S1 **Experimental design for iTRAQ reagent-labeling of CSF sample pools.** Thirty microgram of each pooled group; C2 group (pool-AH1 to AH3), C3 group (pool-IIH1 to IIH2) or WNND, A2 group (poolW1 to W3) were digested with trypsin and the resulting peptides of each sample were specifically labeled with one iTRAQ reagent as indicated below, previously to mix all samples. WNND, West Nile neuroinvasive disease; AH, acute headache; IIH, idiopathic intracranial hypertension; CSF, cerebrospinal fluid.
(DOC)
