# Supplementary material for: Cerebrospinal Fluid Biomarker Candidates Associated with Human WNV Neuroinvasive Disease
Source: PLoS One. 2014 Apr 2;9(4):e93637. doi: 10.1371/journal.pone.0093637 (PMC3973578; doi:10.1371/journal.pone.0093637)
Supplement: Table S1 — Experimental design for iTRAQ reagent-labeling of CSF sample pools. Thirty microgram of each pooled group; C2 group (pool-AH1 to AH3), C3 group (pool-IIH1 to IIH2) or WNND, A2 group (poolW1 to W3) were digested with trypsin and the resulting peptides of each sample were specifically labeled with one iTRAQ reagent as indicated below, previously to mix all samples. WNND, West Nile neuroinvasive disease; AH, acute headache; IIH, idiopathic intracranial hypertension; CSF, cerebrospinal fluid. (DOC) [file pone.0093637.s001.doc]

**Table S1.** Experimental design for iTRAQ reagent-labeling of CSF sample pools. Thirty microgram of each pooled group; C2 group (pool-AH1 to AH3), C3 group (pool-IIH1 to IIH2) or WNND, A2 group (poolW1 to W3) were digested with trypsin and the resulting peptides of each sample were specifically labeled with one iTRAQ reagent as indicated below, previously to mix all samples. WNND, West Nile neuroinvasive disease; AH, acute headache; IIH, idiopathic intracranial hypertension; CSF, cerebrospinal fluid.

| **Sample pools** | **Isobaric iTRAQ**  **reagent** |
| --- | --- |
| AH1 | 113 |
| AH2 | 114 |
| AH3 | 115 |
| IIH1 | 116 |
| IIH2 | 117 |
| W1 | 118 |
| W2 | 119 |
| W3 | 121 |
